# Hearing Function in Spinal and Bulbar Muscular Atrophy (SBMA): A Case Control Study From a Tertiary Referral Center

**DOI:** 10.1111/ene.70213

**Published:** 2025-06-11

**Authors:** Lorenzo Blasi, Leonardo Franz, Alberto Romito, Andrea Fortuna, Giacomo Maria Minicuci, Alen Bebeti, Federica Paredi, Rosario Marchese Ragona, Maria Pennuto, Cosimo de Filippis, Gino Marioni, Gianni Sorarù

**Affiliations:** ^1^ MND Center, Department of Neuroscience DNS University Hospital of Padova Padova Italy; ^2^ Phoniatrics and Audiology Unit, Department of Neuroscience DNS University of Padova Treviso Italy; ^3^ Otolaryngology Section, Department of Neuroscience DNS University of Padova Padova Italy; ^4^ Department of Biomedical Sciences (DBS), university of Padova Padova Italy

**Keywords:** audiometry, case control, hearing, Kennedy disease, SBMA

## Abstract

**Background:**

Expanding on earlier findings of auditory involvement from two small‐scale studies, we conducted a comprehensive evaluation of hearing levels in a larger cohort of SBMA patients.

**Methods:**

Thirty‐six SBMA patients and 36 age‐matched male controls without risk factors for hearing loss underwent a comprehensive audiological assessment, including pure‐tone audiometry at 250, 500, 1000, 2000, 4000, and 8000 Hz frequencies, using both air and bone conduction. The pure‐tone average (PTA) was calculated as the mean threshold at 500, 1000, 2000, and 4000 Hz. A correlation analysis was performed to evaluate the relationship between patients' audiological features and clinical characteristics, including motor disability, as measured by the SBMA functional rating scale (SBMAFRS) and the 6‐min walk test (6MWT).

**Results:**

PTA values were significantly higher in SBMA patients compared to healthy controls (Mann–Whitney *U* test, *p* = 0.0005, and *p* = 0.0001 for the right and left side, respectively), even when analysis was restricted to the 19 SBMA patients without risk factors for hearing loss (Mann–Whitney *U* test, *p* = 0.0148 and *p* = 0.0243 for the right and left ear, respectively). In the latter group, the hearing thresholds of each individual frequency were significantly higher than in controls, except for the intermediate frequencies (2000 Hz on both sides and 1000 on the left one). Negative significant correlations were found between PTA values and both the CAG repeat number and 6MWT distances. Conversely, SBMAFRS scores were overall unrelated to PTA values.

**Conclusions:**

Our findings suggest a disease‐specific hearing impairment in SBMA patients.

## 1 Introduction

Spinal and Bulbar Muscular Atrophy (SBMA, OMIM 313200), also known as Kennedy's disease, is a rare genetic neuromuscular disorder that is characterized by the progressive degeneration of motor neurons in the spinal cord and brainstem, leading to muscle weakness and atrophy [1]. SBMA is caused by CAG/glutamine (polyQ) expansions in the gene coding for androgen receptor (AR) [2]. Expansions to 38 or more repeats cause disease, and the longer the CAG repeat, the more severe the phenotype [3]. The disease fully manifests in males, as they have high levels of androgens, testosterone, and dihydrotestosterone (DHT) in the serum. AR is a transcription factor activated by androgens. In its inactive state, AR resides in the cytosol. Androgen binding results in nuclear translocation, DNA binding, and regulation of the expression of the androgen‐responsive genes [4]. In SBMA, the binding of androgens to the mutated AR protein enhances its toxicity by promoting its nuclear accumulation, causing cell toxicity [4]. In addition to neuromuscular symptoms, SBMA patients show clinical features of androgen insensitivity, which suggests that CAG expansions in AR lead to disease by combining toxic and loss of function mechanisms [5].

Nuclear inclusions of the polyQ‐AR are the pathological hallmark of the disease [6]. However, both nuclear and cytoplasmic accumulation of the mutant AR has been found in residual lower motor neurons, as well as in several neural and non‐neural AR‐expressing tissues in SBMA patients [7]. Accordingly, studies from both humans and animal models have highlighted the pathological and clinical involvement of cell populations other than motor neurons in SBMA [8, 9], including those of the sensory system [10, 11, 12, 13, 14]. Although sensory disturbances in SBMA are clinically subtle and typically limited to a reduction in vibration sense [10], there is well‐documented evidence of both pathological and electrophysiological involvement of primary sensory neurons [7, 10, 11, 12, 13, 14]. Interestingly, Polo et al. [11] also described abnormalities in brainstem auditory evoked potentials (BAEPs) and a sensorineural hearing loss at pure‐tone audiometry in three patients. Increased hearing thresholds and inconsistent BAEPs have been later confirmed in a small patient cohort by [15]. According to ‘The Human Protein Atlas’ [16], AR mRNA‐containing cells are present along the auditory pathway, specifically in the medial geniculate nuclei of the thalamus and in the inferior colliculi of the brainstem. Moreover, the accumulation of mutant AR has been found in both thalamic and pontine nuclei of SBMA patients [7]. Overall, these data suggest a potential impairment of the auditory sensory pathway in SBMA.

The main aim of this investigation has been to report the hearing levels in a series of consecutive patients affected by SBMA. A secondary one has been to compare the hearing function of the sub‐cohort of SBMA patients without risk factors for hearing loss with an age‐matched control group of healthy subjects, as we previously did in other settings [17].

## 2 Methods

### 2.1 Study Population

This study included consecutive patients affected with molecularly defined SBMA who attended the Motor Neuron Disease Clinic at the Neurology Unit, Department of Neuroscience, University of Padova from January 2022 to September 2023. Healthy male subjects without risk factors for hearing loss (as specified in the Clinical Protocol) were selected as controls. These controls were age‐matched with SBMA patients at a 1:1 ratio (Mann–Whitney *U* test, *p* = 0.9301) and were recruited from the Phoniatrics and Audiology Unit, Department of Neuroscience, University of Padova, during the period 2023–2024.

The Local Ethics Committee approved the study (AOP0964) and all study participants provided their informed consent in writing.

### 2.2 Clinical Protocol

Patient characteristics, including age at onset (defined as subjective weakness in any part of the body, whether bulbar and/or spinal), age at the time of audiometry, duration of illness since the onset of weakness, and the number of CAG repeats, were collected. Disease severity was assessed using the Italian version of the Spinal and Bulbar Muscular Atrophy Functional Rating Scale (SBMAFRS) [18], a questionnaire‐based scale measuring physical function in activities of daily living (ADL) [19]. SBMAFRS comprises five main subscales measuring bulbar (5 items), upper limb (2 items), lower limb (2 items), truncal (4 items), and respiratory function (1 item). Specific items within each subscale are scored based on five response options from 0 (worst) to 4 (normal). Additionally, the 6‐min walk test (6MWT), traditionally regarded as the most reliable marker of motor impairment in SBMA [20], was also used. Assistive devices for walking were permitted. Before hearing assessment, all patients were evaluated using a clinical questionnaire focused on personal clinical history, with the emphasis on any evidence of risk factors for hearing loss. These included, in particular, chronic exposure to noise or ototoxic substances including medications, recurrent or chronic ear infections, and previous relevant ear trauma.

Each subject of the study and control population underwent otoscopy and an audiological study including pure‐tone audiometry on the 250–500–1000‐2000‐4000‐8000 Hz frequencies, by both air and bone conduction. The pure‐tone average (PTA) was estimated as the mean threshold value at 500–1000–2000‐4000 Hz.

### 2.3 Statistical Analysis

Continuous variables were summarized by mean and standard deviation for normally distributed variables, and by median and inter‐quartile range (IQR) for non‐normally distributed ones. Categorical variables were synthesized by count and percentage. Mann–Whitney *U* test was applied to non‐normally distributed continuous variables, while Fisher's exact test was applied to the categorical ones. The effect size of comparisons was quantified using Cohen's d. Moreover, to compare the pure‐tone thresholds for each ear between cases and controls, a multivariate model considering possibly heterogeneous covariance matrices, based on the Wald test, was used.

Spearman's rank correlation coefficient was used to test the mutual relationship between different continuous variables. Statistical significance was set at a *p* value < 0.05. All statistical analyses were performed using Stata 16.1 (College Station, TX, USA).

## 3 Results

### 3.1 Clinical Features

A total of 36 SBMA patients were included. Their mean age at disease onset was 45.6 ± 11.1 years (median: 43 years, range: 17–74 years). Mean age at audiometry was 57.9 ± 10.8 years (median: 58.5, range: 34–79 years), after a mean disease duration of 12.27 ± 7.69 years (median: 12 years, range: 1–32 years). The CAG repeat number ranged from 42 to 51 (mean 46.25 ± 2.76).

From a functional viewpoint, the median overall SBMAFRS value was 42.0 (IQR: 37.5–47.0). The partial scores for bulbar, upper limb, trunk, lower limb, and respiratory symptoms have been summarized in Table S1. The mean distance covered by patients during 6MWT was 328.29 ± 155.00 m. Seventeen out of 36 patients had a clinical history of possible exposure to risk factors for sensorineural hearing loss, including noise exposure. None of the patients reported prior use of ototoxic medications.

### 3.2 Audiological Features

The median pure‐tone thresholds for each tested frequency and PTA values have been summarized in Table 1. Considering the whole cohort of 36 SBMA patients, PTA values were significantly higher in SBMA patients compared to controls [21.25 dB (IQR: 15.00–28.75 dB) vs. 13.75 (10.00–20.00) (Mann–Whitney *U* test, *p* = 0.0005), and 23.75 (18.75–30.00) vs. 15.00 (11.25–20.00) (*p* = 0.0001)] for the right and left sides, respectively. The multivariate comparison of pure‐tone thresholds showed significantly higher values for SBMA patients vs. controls (*p* < 0.001 for both right and left sides). A detailed comparison between hearing thresholds in patients and controls for PTA and each tested frequency has been reported in Table 1.

**TABLE 1:** Distribution of audiological variables in the whole cohort of SBMA patients, in the sub‐cohort of SBMA patients without risk factors for hearing loss, and in the matched controls.

Variable	Whole SBMA cohort, median (IQR) mean (SD)	SBMA patients without risk factors, median (IQR), mean (SD)	Matched controls, median (IQR), mean (SD)	Comparison between all SBMA patients and matched controls, Cohen's *d* (95% CI)	Comparison between all SBMA patients and matched controls, *p* value^b^	Comparison between SBMA patients without risk factors and matched controls, *p* value^a^	Comparison between SBMA patients without risk factors and matched controls, Cohen's *d* (95% CI)	Comparison between SBMA patients without risk factors and matched controls, *p* value^b^	Comparison between SBMA patients without risk factors and matched controls, *p* value^a^
Hearing threshold (dB) 250Hz_right	15.00 (15.00–25.00) 19.71 (8.48)	20.00 (15.00–20.00) 19.74 (8.89)	10.00 (10.00–10.00) 10.79 (1.87)	−1.32 (−1.83; −0.81)	**0.0001**	**< 0.0001**	−1.39 (−2.10; −0.67)	**0.0059**	**< 0.0001**
Hearing threshold (dB) 500Hz_right	15.00 (15.00–25.00) 19.00 (8.72)	15.00 (10.00–20.00) 18.42 (9.14)	10.00 (10.00–15.00) 11.58 (2.91)	−1.00 (−1.50; −0.51)		**< 0.0001**	−1.01 (−1.68; −0.33)		**0.0250**
Hearing threshold (dB) 1000Hz_right	15.00 (10.00–25.00) 18.46 (9.59)	15.00 (10.00–20.00) 18.47 (9.97)	10.00 (10.00–10.00) 12.63 (6.53)	−0.61 (−1.08; −0.13)		**0.0001**	−0.69 (−1.34; −0.33)		**0.0030**
Hearing threshold (dB) 2000Hz_right	20.00 (15.00–25.00) 20.29 (9.77)	20.00 (10.00–25.00) 20.79 (10.96)	15.00 (10.00–20.00) 15.79 (7.69)	−0.50 (−0.98; −0.03)		**0.0044**	−0.53 (−1.17; 0.12)		0.1263
Hearing threshold (dB) 4000Hz_right	30.00 (20.00–50.00) 36.86 (20.87)	40.00 (20.00–50.00) 38.95 (20.25)	20.00 (10.00–40.00) 26.32 (18.25)	−0.54 (−1.01; −0.07)		**0.0181**	−0.66 (−1.30; 0.00)		**0.0450**
Hearing threshold (dB) 8000Hz_right	55.00 (20.00–75.00) 48.80 (27.58)	60.00 (25.00–75.00) 55.42 (28.26)	25.00 (10.00–55.00) 32.37 (23.41)	−0.73 (−1.21; −0.25)		**0.0028**	−0.89 (−1.56; −0.25)		**0.0115**
Hearing threshold (dB) PTA_right	21.25 (15.00–28.75) 23.65 (10.12)	21.25 (18.75–28.75) 24.16 (10.86)	13.75 (10.00–20.00) 16.58 (7.52)	−0.73 (−1.21; −0.25)	**0.0031**	**0.0005**	−0.81 (−1.47; −0.14)	**0.0177**	**0.0148**
Hearing threshold (dB) 250Hz_left	15.00 (10.00–20.00) 18.43 (9.53)	15.00 (10.00–20.00) 17.63 (10.05)	10.00 (10.00–10.00) 10.79 (1.87)	−1.10 (−1.60; −0.60)	**0.0001**	**< 0.0001**	−0.95 (−1.61; −0.27)	**0.0019**	**0.006**
Hearing threshold (dB) 500Hz_ left	15.00 (10.00–20.00) 18.57 (10.33)	15.00 (10.00–20.00) 18.68 (11.28)	10.00 (10.00–15.00) 11.84 (3.80)	−0.95 (−1.44; −0.45)		**< 0.0001**	−0.81 (−1.47; −0.14)		**0.0204**
Hearing threshold (dB) 1000Hz_ left	15.00 (10.00–20.00) 17.86 (10.17)	15.00 (10.00–20.00) 17.37 (9.63)	10.00 (10.00–15.00) 13.95 (7.74)	−0.59 (−1.06; −0.11)		**0.0005**	−0.39 (−1.03; −0.25)		0.0668
Hearing threshold (dB) 2000Hz_ left	20.00 (15.00–25.00) 20.86 (10.18)	20.00 (10.00–25.00) 21.05 (9.94)	10.00 (10.00–25.00) 17.89 (12.62)	−0.56 (−1.03; −0.08)		**0.0010**	−0.28 (−0.91; 0.36)		0.1308
Hearing threshold (dB) 4000Hz_ left	45.00 (25.00–60.00) 42.86 (19.86)	40.00 (25.00–65.00) 44.74 (22.33)	20.00 (15.00–40.00) 27.63 (16.68)	−0.86 (−1.35; −0.37)		**0.0011**	−0.87 (−1.53; −0.19)		**0.0190**
Hearing threshold (dB) 8000Hz_ left	55.00 (35.00–65.00) 49.43 (23.03)	60.00 (35.00–70.00) 56.05 (23.72)	25.00 (10.00–50.00) 30.53 (22.10)	−0.90 (−1.39; −0.41)		**0.0003**	−1.11 (−1.79; −0.42)		**0.0016**
Hearing threshold (dB) PTA_ left	23.75 (18.75–30.00) 25.04 (10.27)	25.00 (18.75–30.00) 25.46 (10.74)	15.00 (11.25–20.00) 17.82 (8.76)	−0.94 (−1.42; −0.44)	**0.0002**	**0.0001**	−0.78 (−1.43; −0.11)	**0.0219**	**0.0243**

*Note:* *p*‐values < 0.05, indicating statistical significance, are highlighted in bold.

Abbreviations: 95% CI, 95% confidence interval; IQR, inter‐quartile range; SD, standard deviation.

^a^ Mann–Whitney *U* test.

^b^ Multivariate equality of means test, allowing heterogeneous covariance matrices, using Wald test.

**FIGURE 1:**
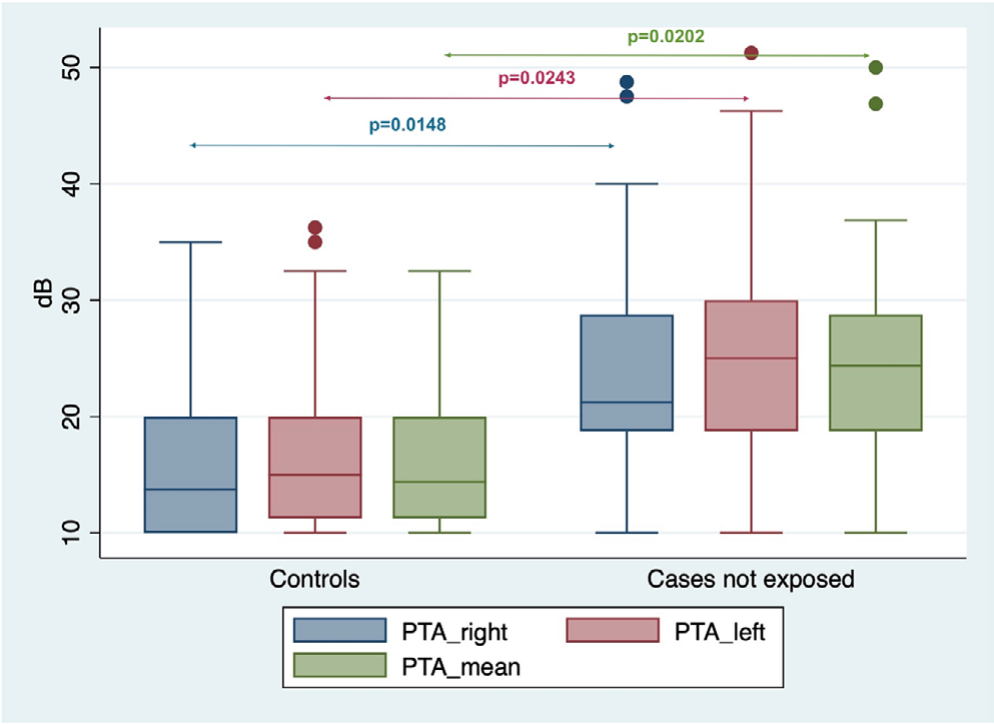
Box plot showing the distribution of PTA values in SBMA patients without risk factors for hearing loss and in sex‐ and age‐matched controls. Blue: Right side PTA; red: Left side PTA; green: Mean PTA. *p* values are based on Mann‐Whiney *U* test.

**FIGURE 2:**
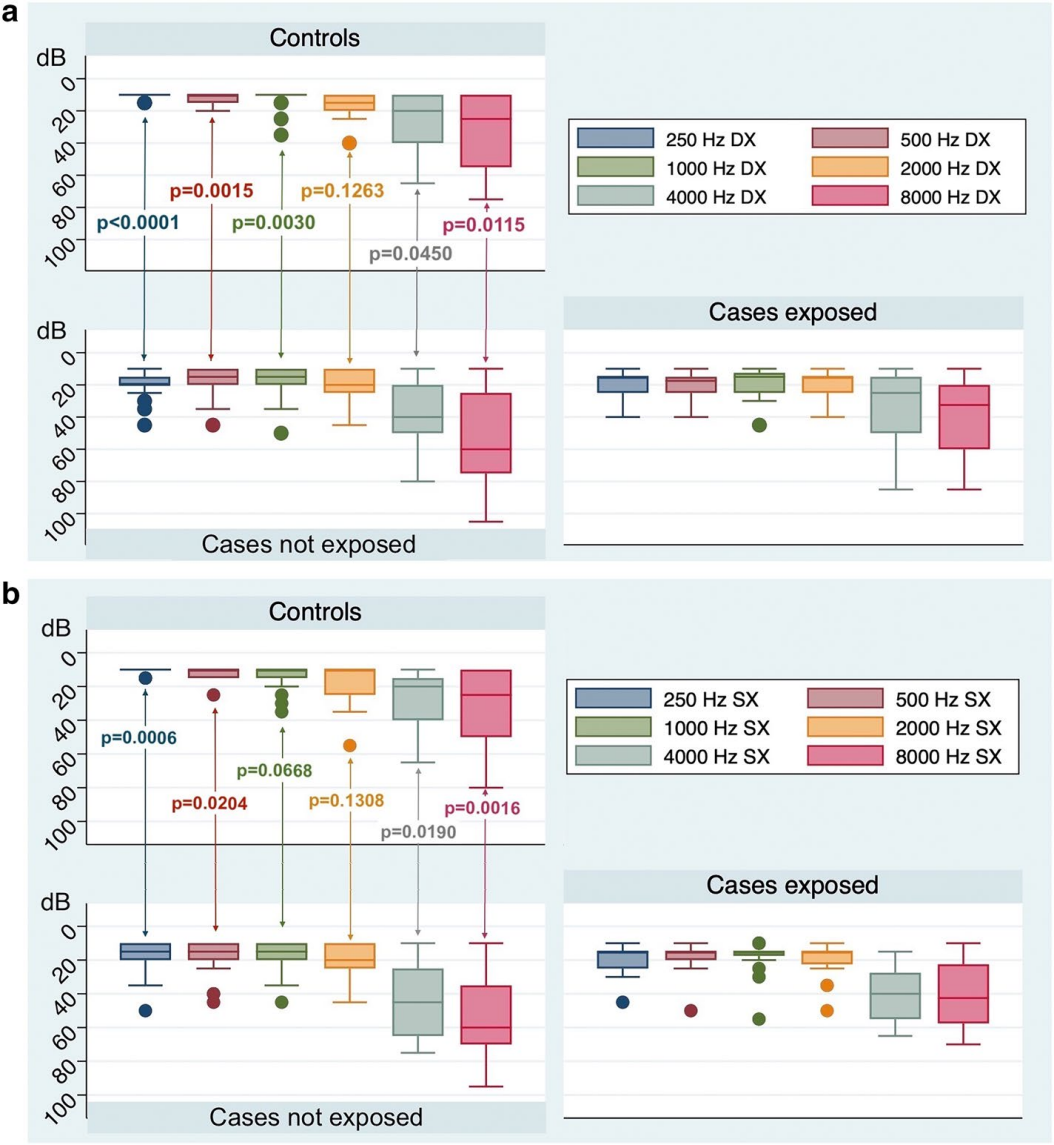
Box plot showing the distribution of hearing threshold values in SBMA patients without risk factors for hearing loss and in sex‐ and age‐matched controls: (a) right side; (b) left side. *p* values are based on Mann‐Whiney *U* test.

Among the 19 SBMA patients without risk factors for hearing loss, the median PTA was 21.25 dB (IQR:18.75–28.75) in the right ear and 25.00 dB (IQR:18.75–30.00) in the left ear. These PTA values were significantly higher than those of controls (Mann–Whitney *U* test, *p* = 0.0148 and *p* = 0.0243 for the right and left ear, respectively; see also Figure 1). Considering each individual tested frequency, the hearing thresholds in SBMA patients with no risk factor for hearing loss were significantly higher than in the healthy controls for 250, 500, 1000, 4000, and 8000 Hz on the right side (Mann–Whitney *U* test, *p* < 0.0001, *p* = 0.0015, *p* = 0.0030, *p* = 0.0450, and 0.0115, respectively; see also Figure 2a), and for 250, 500, 4000, and 8000 Hz on the left side (Mann–Whitney *U* test, *p* = 0.0006, *p* = 0.0204, *p* = 0.0190, *p* = 0.0016, respectively; see also Figure 2b). No significant differences were found in hearing thresholds at 2000 Hz on the right side and at 1000 and 2000 Hz on the left one. The multivariate comparison of pure‐tone thresholds showed significantly higher values for SBMA patients without risk factors for hearing loss vs. controls (*p* = 0.0059 and *p* = 0.0019 for the right and left sides respectively).

### 3.3 Analysis of the Correlation Between Audiological and Clinical Features

**FIGURE 3:**
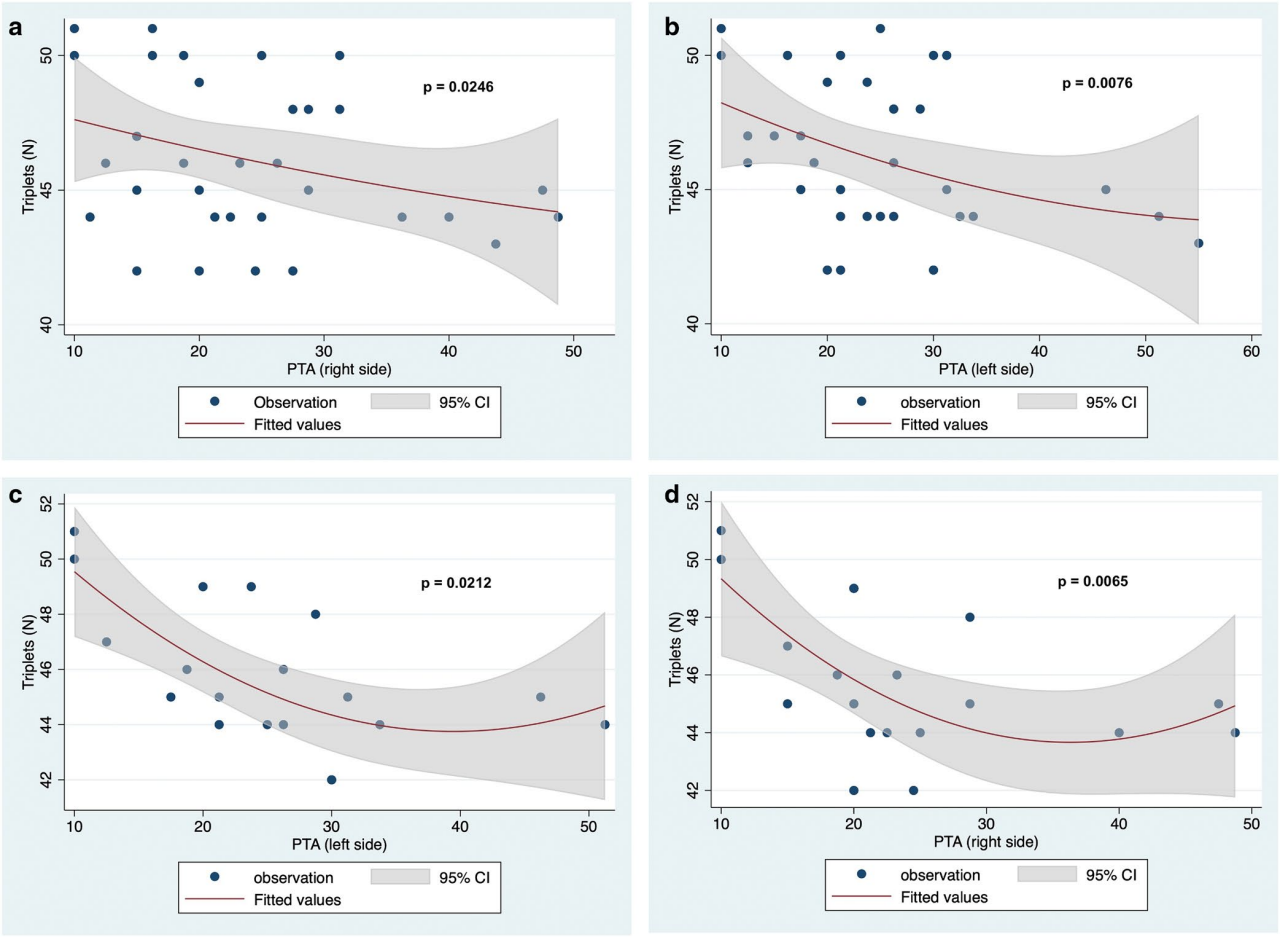
Correlation between number of CAG repeats and PTA values: (a) right side, entire cohort of SBMA patients; (b) left side, entire cohort of SBMA patients; (c) right side, SBMA patients without risk factors for hearing loss; (d) left side, SBMA patients without risk factors for hearing loss. *p* values are based on the Spearman correlation model.

**FIGURE 4:**
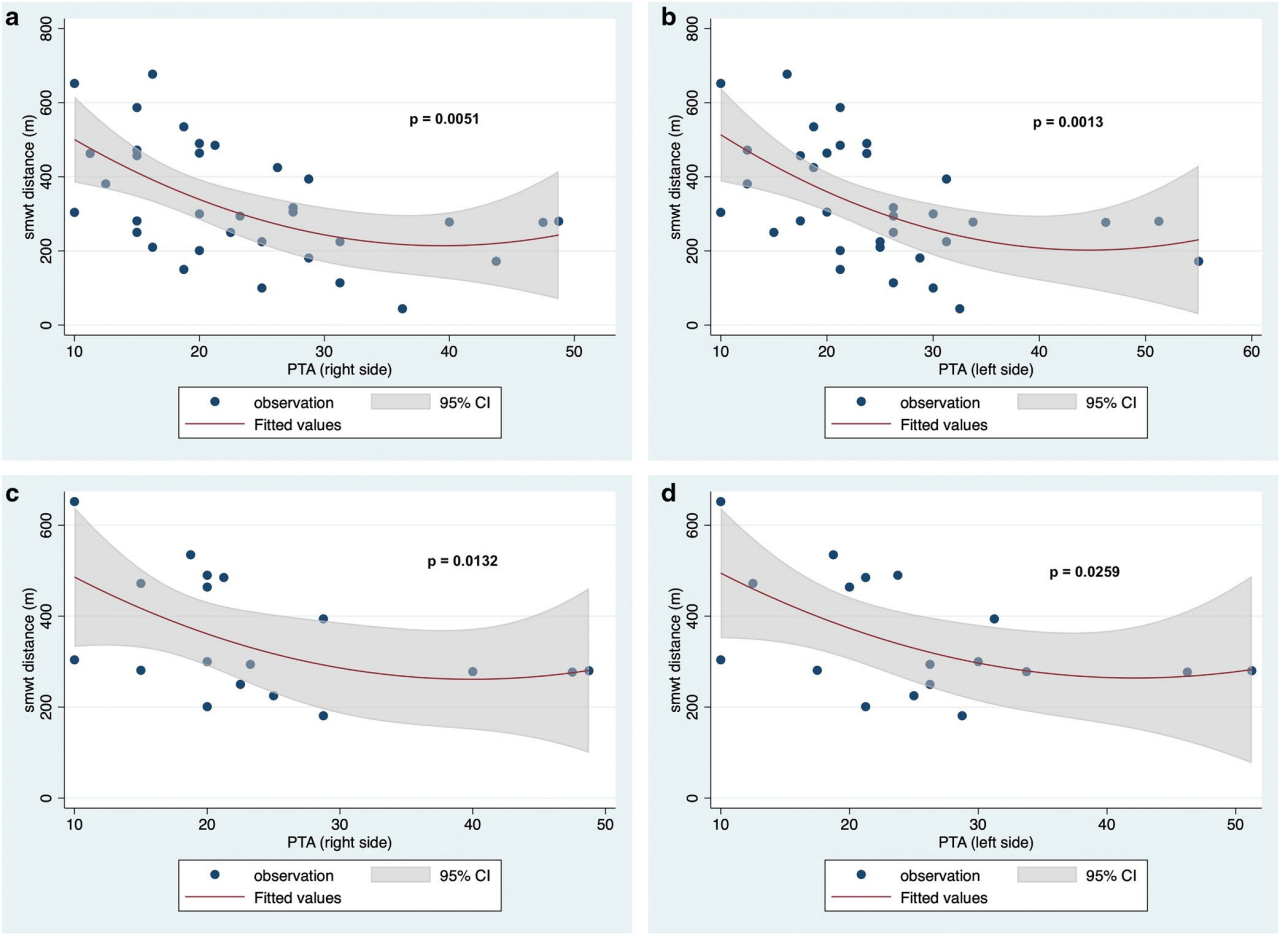
Correlation between distance at six‐meter walking test and PTA values: (a) right side, entire cohort of SBMA patients; (b) left side, entire cohort of SBMA patients; (c) right side, SBMA patients without risk factors for hearing loss; (d) left side, SBMA patients without risk factors for hearing loss. *p* values are based on the Spearman correlation model.

Across the entire cohort of SBMA patients, PTA values were found to be positively correlated with age at the time of audiometry (Spearman's rank correlation model: rho = 0.5389, *p* = 0.0018; and rho = 0.6180; *p* = 0.0002 for PTA on the right and left sides, respectively), but not with disease duration. Additionally, PTA values negatively correlated with the number of CAG repeats (rho = −0.4030, *p* = 0.0246; and rho = −0.4702, *p* = 0.0076 for PTA on the right and left sides, respectively; see also Figure 3a,b) and the 6MWT (rho = −0.4905, *p* = 0.0051; and rho = −0.5522, *p* = 0.0013 for PTA on the right and the left side, respectively, see also Figure 4a,b). On the other hand, no correlations were disclosed between PTA values and the overall SBMAFRS scores, although significant or nearly significant negative correlations were found for the lower limb SBMAFRS scores (rho = −0.2579, *p* = 0.0123; and rho = −0.2151, *p* = 0.0507 for PTA on the left and right ear, respectively) and for the trunk (rho = −0‐3761, *p* = 0.0371, and rho = −0.3208, *p* = 0.0785 for PTA on the left and right ear, respectively) subscales. A detailed breakdown of the correlations found between the considered audiological and clinical variables in the whole Kennedy disease series has been reported in Table S2.

Analyzing only the sub‐cohort of SBMA patients without risk factors for hearing loss, the positive correlation between PTA values and age retained its statistical significance (Spearman's rank correlation model: rho = 0.5827, *p* = 0.0112; and rho = 0.6494, *p* = 0.0035 for PTA on the right and left side, respectively). Accordingly, the significant negative correlations between PTA values and the number of triplets (rho = −0.5383, *p* = 0.0212, and rho = −0.6159, *p* = 0.0065 for PTA on the right and the left side, respectively, see also Figure 3c,d), or 6MWT (rho = −0.5714, *p* = 0.0132, and rho = −0.5230, *p* = 0.0259 for PTA on the right and the left side, respectively, see also Figure 4c,d) were found in the subgroup of patients with no exposure to risk factors. Table S3 summarizes the correlations between audiological and clinical variables in this subgroup.

## 4 Discussion

Emerging evidence suggests that SBMA is a multisystem syndrome rather than solely a neuromuscular disorder [8, 9]. Building on previous observations of auditory involvement in two separate small series [11, 15], our study evaluated hearing levels in a larger cohort of SBMA patients.

The primary finding was a significantly higher threshold across all frequencies in SBMA patients compared to age‐matched controls. This difference remained significant even when analysis was limited to the subgroup of patients without exposure to acoustic risk factors, although it was not observed at the intermediate frequencies (1000–2000 Hz). Additionally, it was found that the elevation in auditory thresholds correlated with patients' age. While these data might suggest the para‐physiological characteristic of presbycusis, the audiometric frequency pattern observed in patients argues against this interpretation. Indeed, the audiometric curve of SBMA patients exhibited an ascending‐descending pattern, whereas the control group displayed a sloping pattern in the high‐frequency range, which is typical of age‐related hearing loss. Furthermore, the overall PTA values for the SBMA patients were significantly higher than those of the sex‐ and age‐matched controls.

Another noteworthy finding of our investigation is that the hearing deficit was more pronounced in SBMA patients with a lower number of CAG repeats. This supports existing literature indicating that in SBMA, the sensory system is differently affected compared to the motor system. Indeed, neurophysiological studies have shown that peripheral sensory pathways are more compromised with shorter CAG repeats, whereas motor pathways are more affected with longer CAG repeats [13, 21]. This phenomenon may be attributed to the differential accumulation of pathological AR in motor and sensory neurons [7]. Mutant AR accumulates diffusely within the nuclei of spinal motor neurons, whereas cytoplasmic aggregation predominates in sensory neurons [7]. The extent of diffuse intranuclear accumulation in motor neurons is closely correlated with the size of the CAG repeats [7]. Conversely, cytoplasmic aggregation of mutant AR has been more frequently noted in patients with shorter CAG repeats [7]. The presence of cytoplasmic polyQ‐AR in neurons of the auditory pathway has not been extensively investigated [7]. However, the observation of prominent cytoplasmic inclusions in dorsal root ganglion neurons suggests the possibility of similar findings in the cochlear ganglia.

The final important result from this study stems from the correlation analysis of hearing impairment with disability measures. We observed no substantial relationship between auditory thresholds and motor function, as measured by the SBMAFRS. This finding supports the differential involvement of motor and sensory neurons in SBMA, further emphasizing that the pathogenic mechanisms leading to sensory symptoms, including hearing impairment, may be independent from those involved in the neuromuscular clinical presentation. Conversely, we identified a robust negative correlation between 6MWT values and hearing function, indicating that patients who walked less had higher auditory thresholds. Although this finding appears contradictory to the earlier reasoning, it interestingly aligns with the weak correlation noted for the SBMAFRS items exploring lower limb activities, i.e., walking and climbing stairs, with PTA values. It could be postulated that SBMA patients with greater auditory involvement may demonstrate increased instability and related difficulties in walking, which cannot be fully explained by muscle weakness alone. Generally speaking, the relationship between sensorineural hearing loss and the vestibular system is complex and not clearly understood [22]. It has been reported that, although different, there seems to be a similar functioning between the vestibular and auditory systems, and when one is dysfunctional, the other may also present some degree of impairment [23]. In a retrospective cross‐sectional study, the association between hearing and postural balance was evaluated [24]: increased hearing threshold was an independent predictor of increased postural instability. Nonetheless, it was also to consider that the 6MWT assesses a global and integrated response from all systems engaged during exercise, including the pulmonary and cardiovascular systems, systemic and peripheral circulation, in addition to neuromuscular function [25]. Although this is beyond the scope of the present study, it will be very intriguing and potentially clinically relevant to investigate the association between SBMA, hearing, and balance control with modern available tools.

To the best of our knowledge, the present study is the first to have examined hearing function in a relatively large series of SBMA patients and to have investigated how hearing function differed according to patient characteristics and function. Of value, the SBMA cohort was compared to an age‐matched control group of healthy male subjects. However, given the exploratory nature of the study, we did not further characterize the auditory deficit, particularly by conducting BAEPs or other assessments, nor did we evaluate the relationship between auditory thresholds and concurrent motor and sensory conduction studies.

Hearing impairment is a significant cause of disability in the general population and the odds of falling is 2.39 times greater among older adults with hearing loss than older adults with normal hearing [26]. Therefore, hearing loss might represent for SBMA patients an adjunctive risk factor for falling along with motor weakness and peripheral sensory disturbances. Last but not least, it is worth reminding that an independent association between hearing loss and cognitive decline is also well‐established [27]. Thus, we believe that our study findings call for further investigation in a larger SBMA cohort.

## Author Contributions

**Lorenzo Blasi:** investigation. **Leonardo Franz:** investigation, formal analysis. **Alberto Romito:** investigation. **Andrea Fortuna:** investigation. **Giacomo Maria Minicuci:** investigation. **Alen Bebeti:** investigation. **Federica Paredi:** investigation. **Rosario Marchese Ragona:** investigation. **Maria Pennuto:** writing – review and editing. **Cosimo de Filippis:** writing – review and editing. **Gino Marioni:** conceptualization, writing – original draft. **Gianni Sorarù:** conceptualization, funding acquisition, writing – review and editing, supervision, writing – original draft.

## Conflicts of Interest

The authors declare no conflicts of interest.

## Supporting information


Data S1.

## Data Availability

The data that support the findings of this study are available from the corresponding author upon reasonable request.
