# Supplementary material for: Hearing Function in Spinal and Bulbar Muscular Atrophy (SBMA): A Case Control Study From a Tertiary Referral Center
Source: Eur J Neurol. 2025 Jun 11;32(6):e70213. doi: 10.1111/ene.70213 (PMC12152368; doi:10.1111/ene.70213)
Supplement: Supplementary file 1 — Data S1. [file ENE-32-e70213-s001.docx]

***Hearing function in Spinal and Bulbar Muscular Atrophy (SBMA): a case control study from a tertiary referral center.***

*Blasi Lorenzo^1^* , Franz Leonardo^2^* , Romito Alberto^1^, Fortuna Andrea^1^, Minicuci Giacomo Maria^1^, Bebeti Alen^1^, Paredi Federica^1^, Marchese Ragona Rosario^3^, Maria Pennuto^4^, de Filippis Cosimo^2^, Marioni Gino^2^ and Sorarù Gianni^1^*

1- MND Center, Department of Neuroscience DNS, University Hospital of Padova, Padova, Italy;

2- Phoniatrics and Audiology Unit, Department of Neuroscience DNS, University of Padova, Treviso, Italy;

3- Otolaryngology Section, Department of Neuroscience DNS, University of Padova, Padova, Italy

4- Department of Biomedical Sciences (DBS), University of Padova, 35131 Padova, Italy.

* These authors contributed equally to the manuscript realization

Correspondence to:

- Gianni Sorarù, MND Center, University Hospital of Padova, via Giustiniani 2, 35128 Padova, Italy; [gianni.soraru@unipd.it](mailto:gianni.soraru@unipd.it)

- Gino Marioni, Phoniatrics and Audiology Unit, Department of Neuroscience DNS, University of Padova, Treviso Hospital, piazzale dell’Ospedale 1, 31100 Treviso, Italy

[gino.marioni@unipd.it](mailto:gino.marioni@unipd.it)

**Supplemental Tables**

**Supplemental table 1** Overall and partial SBMAFRS scores for bulbar, upper limb, trunk, lower limb, and respiratory symptoms. *IQR: inter-quartile range*.

| **SBMAFRS scores** | **Median (IQR)** |
| --- | --- |
| **Bulbar symptoms score** | 15.0 (13.5-16.5) |
| **Upper limb symptoms score** | 6.5 (4.5-8.0) |
| **Trunk symptoms score** | 12.0 (11.0-15.0) |
| **Lower limb symptoms score** | 4.0 (3.0-6.0) |
| **Respiratory symptoms score** | 4.0 (3.0-4.0) |
| **Total SBMAFRS score** | 42.0 (37.5-47.0) |

**Table Supplemental 2** Correlations between clinical and audiological variables in the entire cohort of SBMA patients (gray cells: Spearman’s rho coefficients; white cells: p-values). *PTA: pure-tone average; N: number; 6mwt: six-minute walking test; SBMAFRS: Spinal and Bulbar Muscular Atrophy Functional Rating Scale.*

|  | **PTA_right** | **PTA_left** | **Age** | **N of triplets** | **Disease duration** | **6mwt** | **SBMAFRS tot** | **SBMAFRS_ bulbar** | **SBMAFRS_upper limbs** | **SBMAFRS_**  **trunk** | **SBMAFRS_**  **lower limbs** | **SMAFRS_**  **respiratory** |
| --- | --- | --- | --- | --- | --- | --- | --- | --- | --- | --- | --- | --- |
| **PTA_right** |  | 0.8951 | 0.5389 | -0.4030 | 0.0980 | -0.4905 | -0.3178 | -0.1970 | -0.2151 | -0.3208 | -0.3540 | -0.2491 |
| **PTA_left** | <0.0001 |  | 0.6180 | -0.4702 | 0.1613 | -0.5522 | -0.3483 | -0.1914 | -0.2579 | -0.3761 | -0.4440 | -0.2244 |
| **Age** | 0.0018 | 0.0002 |  | -0.5302 | 0.2627 | -0.5559 | -0.4317 | -0.3233 | -0.3631 | -0.3472 | -0.4852 | -0.1070 |
| **N of triplets** | 0.0246 | 0.0076 | 0.0022 |  | 0.3347 | 0.0828 | 0.1303 | 0.2110 | 0.1434 | -0.0133 | 0.0945 | 0.1421 |
| **Disease duration** | 0.6000 | 0.3859 | 0.1533 | 0.0657 |  | -0.6152 | -0.4063 | -0.0675 | -0.4087 | -0.5709 | -0.5742 | 0.0581 |
| **6mwt** | 0.0051 | 0.0013 | 0.0012 | 0.6579 | 0.0002 |  | 0.6426 | 0.2226 | 0.6007 | 0.7447 | 0.7651 | 0.1619 |
| **SBMAFRS tot** | 0.0815 | 0.0548 | 0.0153 | 0.4849 | 0.0233 | 0.0001 |  | 0.8065 | 0.8377 | 0.9122 | 0.8381 | 0.5496 |
| **SBMAFRS_ bulbar** | 0.2882 | 0.3023 | 0.0760 | 0.2545 | 0.7182 | 0.2287 | <0.0001 |  | 0.5867 | 0.6072 | 0.5835 | 0.6798 |
| **SBMAFRS_upper limbs** | 0.2453 | 0.1613 | 0.0447 | 0.4416 | 0.0224 | 0.0004 | <0.0001 | 0.0005 |  | 0.7579 | 0.6758 | 0.2994 |
| **SBMAFRS_**  **trunk** | 0.0785 | 0.0371 | 0.0557 | 0.9435 | 0.0008 | 0.0000 | <0.0001 | 0.0003 | <0.0001 |  | 0.8581 | 0.3696 |
| **SBMAFRS_**  **lower limbs** | 0.0507 | 0.0123 | 0.0057 | 0.6130 | 0.0007 | 0.0000 | <0.0001 | 0.0006 | <0.0001 | <0.0001 |  | 0.3311 |
| **SMAFRS_**  **respiratory** | 0.1766 | 0.2250 | 0.5667 | 0.4458 | 0.7562 | 0.3842 | 0.0014 | <0.0001 | 0.1018 | 0.0407 | 0.0689 |  |

**Supplemental table 3.** Correlations between clinical and audiological variables in the sub-cohort of SBMA patients without risk factors for hearing loss (gray cells: Spearman’s rho coefficients; white cells: p-values). *PTA: pure-tone average; N: number; 6mwt: six-minute walking test; SBMAFRS: Spinal and Bulbar Muscular Atrophy Functional Rating Scale.*

|  | **PTA_right** | **PTA_left** | **Age** | **N of triplets** | **Disease duration** | **6mwt** | **SBMAFRS tot** | **SBMAFRS_ bulbar** | **SBMAFRS_upper limbs** | **SBMAFRS_**  **trunk** | **SBMAFRS_**  **lower limbs** | **SMAFRS_**  **respiratory** |
| --- | --- | --- | --- | --- | --- | --- | --- | --- | --- | --- | --- | --- |
| **PTA_right** |  | 0.9319 | 0.5827 | -0.5383 | 0.0412 | -0.5714 | -0.4047 | -0.1557 | -0.3250 | -0.4074 | -0.2966 | -0.2573 |
| **PTA_left** | <0.0001 |  | 0.6494 | -0.6159 | 0.0405 | -0.5230 | -0.3995 | -0.1491 | -0.3825 | -0.3748 | -0.3386 | -0.3124 |
| **Age** | 0.0112 | 0.0035 |  | -0.7355 | -0.0866 | -0.5493 | -0.4785 | -0.2260 | -0.4257 | -0.3839 | -0.3252 | -0.2793 |
| **N of triplets** | 0.0212 | 0.0065 | 0.0005 |  | 0.2519 | 0.4271 | 0.3708 | 0.2351 | 0.5152 | 0.3255 | 0.2703 | 0.2225 |
| **Disease duration** | 0.8710 | 0.8733 | 0.7327 | 0.3133 |  | -0.4104 | -0.3578 | 0.0831 | -0.3452 | -0.4945 | -0.4863 | 0.0737 |
| **6mwt** | 0.0132 | 0.0259 | 0.0182 | 0.0771 | 0.0907 |  | 0.6411 | 0.0791 | 0.4727 | 0.7043 | 0.6898 | 0.2136 |
| **SBMAFRS tot** | 0.0957 | 0.1005 | 0.0446 | 0.1298 | 0.1449 | 0.0041 |  | 0.7242 | 0.7625 | 0.9140 | 0.8241 | 0.5256 |
| **SBMAFRS_ bulbar** | 0.5374 | 0.5548 | 0.3671 | 0.3478 | 0.7430 | 0.7550 | 0.0007 |  | 0.4797 | 0.5260 | 0.4516 | 0.6227 |
| **SBMAFRS_upper limbs** | 0.1881 | 0.1172 | 0.0782 | 0.0287 | 0.1606 | 0.0476 | 0.0002 | 0.0440 |  | 0.6572 | 0.6314 | 0.2565 |
| **SBMAFRS_**  **trunk** | 0.0933 | 0.1254 | 0.1158 | 0.1875 | 0.0370 | 0.0011 | <0.0001 | 0.0249 | 0.0030 |  | 0.8494 | 0.3297 |
| **SBMAFRS_**  **lower limbs** | 0.2321 | 0.1693 | 0.1880 | 0.2781 | 0.0407 | 0.0015 | <0.0001 | 0.0599 | 0.0049 | <0.0001 |  | 0.2090 |
| **SMAFRS_**  **respiratory** | 0.3027 | 0.2070 | 0.2618 | 0.3748 | 0.7714 | 0.3948 | 0.0251 | 0.0058 | 0.3043 | 0.1816 | 0.4053 |  |
